# GENDER DIFFERENCES IN THE RELATIONSHIP BETWEEN POLYPHARMACY AND DEPRESSIVE SYMPTOMS AMONG OLDER ADULTS

**DOI:** 10.1093/geroni/igae098.0517

**Published:** 2024-12-31

**Authors:** Xiao Qiu, Ziyao Xu, Na Sun, Cassandra Hua, J Scott Brown

**Affiliations:** Miami University, Oxford, Ohio, United States; Miami University, Oxford, Ohio, United States; Miami University, Oxford, Ohio, United States; University of Massachusetts Lowell, Lowell, Massachusetts, United States; Miami University, Oxford, Ohio, United States

## Abstract

Polypharmacy is prevalent among older adults and is associated with increased depressive symptoms, yet differences in the relationship by gender are not well known. Understanding these gender-specific differences is crucial for developing targeted interventions to address depressive symptoms among older adults. This study examines the relationship between polypharmacy and depressive symptoms among US community-dwelling older adults aged 65 or older (n = 3,143), utilizing data from the 2006 and 2008 waves of the Health and Retirement Study (HRS) core survey and its supplementary drug surveys. Employing negative binomial generalized estimating equation (GEE) models, polypharmacy is defined as the concurrent use of five or more prescription medications, while depressive symptoms are assessed using the 8-item version of the Center for Epidemiologic Studies Depression (CESD-8). Over 40% of respondents aged 65 or older from the 2006 and 2008 waves regularly use five or more prescription medications. Polypharmacy is associated with increased depressive symptoms (CIRR=1.20, 95% CI= 1.15 -1.25). Notably, the relationship between polypharmacy and depressive symptoms varies by gender. Among women, polypharmacy is linked to a lower number of depressive symptoms (AIRR = 0.90, 95% CI = 0.84 - 0.95), whereas among men, it is associated with an increased number (AIRR =1.12, 95% CI = 1.05 -1.19). These findings highlight the importance of tailored interventions and healthcare strategies.

